# Defining what a ‘carer’ is and the role they play in in-patient mental healthcare: A focus group study with patients, carers and clinicians

**DOI:** 10.1192/bjo.2020.70

**Published:** 2020-08-18

**Authors:** Aysegul Dirik, Justina Kaselionyte, Domenico Giacco, Stefan Priebe

**Affiliations:** Unit for Social & Community Psychiatry, (WHO Collaborating Centre for Mental Health Services Development), Queen Mary University of London, UK; Unit for Social & Community Psychiatry, (WHO Collaborating Centre for Mental Health Services Development), Queen Mary University of London, UK; Unit for Social & Community Psychiatry, (WHO Collaborating Centre for Mental Health Services Development), Queen Mary University of London, UK; Unit for Social & Community Psychiatry, (WHO Collaborating Centre for Mental Health Services Development), Queen Mary University of London, UK

**Keywords:** Carers, inpatient treatment, focus groups

## Abstract

**Background:**

The value of carer involvement has been extensively researched and promoted. However, the field lacks exploration of conceptual issues, which might help to explain why there are widespread difficulties in putting policy into practice in this area, as implementation rates remain low internationally.

**Aims:**

This qualitative study explored patients’, carers’ and clinicians’ perspectives on the role of carers in mental healthcare, particularly with regards to in-patient settings.

**Method:**

Sixteen focus groups were conducted with patients, carers and clinicians who have current or previous experience of in-patient settings. A thematic analysis was conducted on the transcripts, exploring two key domains: (a) what a ‘carer’ is, and (b) how the ‘carer’ role is described within the context of the hospital environment.

**Results:**

Participants diverged in their opinions of what the ‘carer’ role entails, and the perceived helpfulness of it. Issues unique to the in-patient setting were identified, such as the role of the hospital environment in enabling or being a barrier to carer involvement. These differing perspectives and contextual factors had an impact on the position of carers in the hospital setting, as they could be viewed as helpful, a hindrance or as passive visitors, depending on the perspectives of clinicians.

**Conclusions:**

More clarity and agreement is needed between patients, carers and clinicians in terms of how the ‘carer’ role is defined. This has the potential to improve carers’ experience of involvement in hospital settings.

## Background

In the field of mental healthcare, the involvement of family and friends, often referred to as ‘caregivers’ or ‘carers’, has been demonstrated to be useful for a variety of outcomes for patients. This includes a decrease in relapse and rates of readmission to hospital.^1^ The efficacy and implementation of carer involvement has therefore received much attention in research and carer involvement policies and guidelines are widely promoted in mental healthcare internationally (for example from the UK National Collaborating Centre for Mental Health^2^ and UK Department of Health and Social Care^3^). However, the literature lacks an examination of fundamental conceptual issues,^4^ the exploration of which might help us to better understand why carer involvement is so widely promoted in theory yet so poorly or inconsistently implemented in practice, both in the UK and internationally.^5,6^

‘Carer’ is a policy-derived term, which aims to value the contribution that family members and friends make to patients’ care. However, there is a need to explore how the ‘carer’ role is actually understood by patients, their family members and friends and front-line clinicians. This is especially pertinent in hospital settings, as patients may be admitted to hospital following the deterioration of relationships or as a result of a breakdown in their usual social support networks.^7^ There is therefore value in exploring this topic to better understand how ‘carers’ are viewed in the context of mental healthcare, and how this might have an impact on the nature of their involvement in patients’ care.

## Research question

How do patients, carers and clinicians conceptualise the role of carers in the context of mental healthcare and in-patient settings?

## Method

### Design

Focus groups were conducted with patients, carers and clinicians to explore their opinions and experiences of carer involvement in in-patient mental healthcare. The groups were conducted as part of a larger study of stakeholders’ views on what carer involvement in in-patient mental healthcare should entail, and the methodology is described in further detail in a separate article.^8^ For the present part of the study, the topic guide contained questions and prompts pertaining to participants’ conceptualisations of what the carer role entails, and the use of the term ‘carer’.

### Participants

Purposive sampling was used to recruit patients and carers through the East London NHS Foundation Trust in-patient and out-patient mental health services as well as local service user and carer organisations located in the London Boroughs of Hackney, Newham and Tower Hamlets. Advertisements were put on social media (Twitter and Facebook). All clinicians who participated in the study worked in in-patient services at the East London NHS Foundation Trust.

Maximum variation sampling was used to ensure a diverse sample of participants across mental health settings (acute in-patient units, triage wards, community settings), local boroughs and, in the case of staff, job positions. Patient and carer participants were recruited both from settings where a patient was currently in hospital, and from those who had been admitted to hospital within the past 5 years. Additionally, carers were recruited from both settings where an individual might already self-identify as a ‘carer’ (for example carer organisations) and settings where they might not do so (for example by being approached by a clinician when they visit a relative in hospital). Carers could also self-refer by responding to adverts shared widely via email lists, Twitter and bulletin boards. The advert did not use the word ‘carer’ but instead asked ‘have you or someone you support ever been admitted to hospital for mental health reasons?’. This was to attract participants with potentially wide-ranging views on the ‘carer’ role, as well as those who were not familiar with the term ‘carer’.

Sample size was initially planned on the basis of aiming for 6–8 participants per focus group, and 6–12 groups in total, as recommended by Finch, Lewis & Turley (cited in Ritchie et al).^9^ This was envisaged to allow for active participation for all members while giving individuals opportunities for more detailed discussion. However, adjustments were made for each group type, for example carers and clinicians were invited in higher numbers, to allow for the possibility that a proportion would cancel because of caring or working responsibilities.

Patients over 18 years old who were able to provide informed consent, had experience of admission to psychiatric hospital (within 5 years) and sufficient command of English were eligible for the study. Carers were eligible if they were over 18 years old, had experience of supporting someone who was admitted to psychiatric hospital (within 5 years) and sufficient command of English. Clinicians had to have current experience of working in acute in-patient settings.

Potentially eligible patients who were identified by clinicians were asked permission to receive further information from researchers about the study. Patients, carers and clinicians who self-referred were also given the opportunity to discuss details of the study with the researcher, before arranging to attend a focus group.

### Ethics statement

All procedures contributing to this work comply with the ethical standards of the relevant national and institutional committees and with the Helsinki Declaration of 1975, as revised in 2008. Ethical and institutional approvals were provided by the East London NHS Foundation Trust and the East of England – Essex NHS Research Ethics Committee (ref:15/EE/0456). All participants provided their written informed consent to take part in the study, as well as providing verbal consent to begin recording.

### Procedures

Focus groups were facilitated by two researchers, with at least one clinically experienced and able to provide support if any of the participants became distressed or agitated. Except for one mixed patient–carer group, separate focus groups were conducted with carers, patients and clinicians to account for possible counterproductive dynamics between the groups and ensure that each group could express their views freely. Although up to ten participants were sought for most of the focus groups, smaller groups of three to five participants were arranged with patients who were currently in hospital, to minimise overstimulation and allow each participant to share their opinions comfortably.

A topic guide was developed based on guidance in Ritchie et al^9^ and was designed to facilitate up to 90 minutes of discussion. Each of the three participant groups had a slightly amended version of the same topic guide, so that the questions were relevant to them as patients, carers or clinicians. The initial aim of the guide was to gather opinions to develop a carer involvement intervention to be used in in-patient settings. After introductions and ground-rule setting, two main topics were introduced, each with a set of questions, probes and prompts to encourage active participation of all members. The discussion topics began (a) generally, asking participants about their views on carer involvement in mental healthcare, and (b) developed into specific questions about how to involve carers in hospital settings, including a discussion of barriers and facilitators to involvement in different clinical procedures. The topic guide was amended after five focus groups, as the questions on hospital procedures and potential interventions were refined following discussions in the initial groups.

The guide was applied flexibly to enable open discussions within each group. Consequently, although the whole topic guide was covered in each group, different groups discussed some topics more in-depth than others. Participants were asked open-ended questions to stimulate discussions, and all questions were posed as neutrally as possible to encourage them to express their honest opinions. For example, they were asked what they think about the term ‘carer’ and following this, they were asked to discuss what the helpful and unhelpful aspects might be of involving carers in in-patient treatment.

### Analysis

The focus groups were audio recorded and transcribed using intelligent verbatim and omission of identifiable information, such as names. The analysis was conducted independently of the linked study,^8^ which had focused on the practicalities of barriers and facilitators to carer involvement in in-patient treatment. NVivo software was used for coding and organising data during the analysis. Interim analysis was conducted by J.K. and A.D. after nine focus groups and a decision was made to continue with recruitment. After 16 focus groups, J.K., A.D. and D.G. had a reflective discussion about whether saturation of themes had been reached, and it was decided to cease recruitment.

Inductive thematic analysis^10^ was used to analyse the transcripts. This involved looking for common themes and exploring whether there are any similarities and differences between the participant groups. The stages included initial familiarisation with the data corpus by reading all transcripts. Initial coding of the transcripts was then conducted for all transcripts with the research question in mind. Distinct areas of exploration were then identified through the initial coding, and further analysis was conducted within the framework of two domains: (a) what a ‘carer’ is, and (b) how the ‘carer’ role is described within the context of the hospital environment.

An iterative process of identifying subthemes through reflective discussions was conducted throughout the analysis. Labels were attached to each quote during the coding process to identify whether it came from a patient, carer or clinician. Comparisons between the three participant groups were then made at later stages of analysis, once initial themes had been formed. Clusters of related subthemes were converted to overarching themes, with transcripts and quotes being re-checked to ensure consistency of the themes. For example, initial coding of quotes describing ‘battles’ were further analysed to understand which aspect of the hospital context and/or procedures was resulting in this perception, and what this meant in terms of being a ‘carer’. Largely, these quotes described how carers were perceived by professionals as being ‘difficult’ and there were several examples from different participant groups to support this view. These quotes were placed into themes after the transcripts were checked for alternative views. After finalising the themes, quotes were extracted for the article for transparency and to illustrate the findings, although some demographic information was changed to preserve anonymity.

All transcripts were independently coded and analysed by A.D., a researcher with a primary interest in patient perspectives on family involvement and J.K., a researcher with interests in sociocultural perspectives on mental health. The findings were discussed and agreed with a third researcher (D.G.), an academic and clinical psychiatrist who had also conducted some of the focus groups and was familiar with the transcripts. These backgrounds may have influenced each author's personal interpretation of the themes. However, efforts were made to maximise the rigour and trustworthiness of the analysis process by analysing the transcripts separately and then having multiple reflective discussions on the development of the themes. Any points of disagreement were discussed and the transcripts were re-checked throughout the analysis process before deciding on the final themes.

## Results

### Participants

Eighty-six participants attended 16 focus groups held between 2014 and 2016. This included 31 patients, 22 carers and 33 clinicians. Four focus groups were held with carers, five with patients, six with clinicians. Clinician focus groups were broadly separated by profession: ward managers, nurses, psychologists, psychiatrists and support workers. One mixed group was held with both patients and carers. Smaller groups were held for participants currently in acute treatment to support their participation; the size of all groups varied from three to ten participants. Sociodemographic characteristics of the participants have been provided in Table 1. All participants had experience of voluntary or involuntary admission to a psychiatric hospital within the past 5 years, either as a patient, carer or staff member. Further demographic details about the participants are available in the linked study.^8^

**Table 1:** Sociodemographic characteristics of participants

	Patients	Carers	Clinicians
Gender, *n*			
Men	16	2	16
Women	15	20	17
Age			
Mean (s.d.)	43 (12.3)	51 (15.8)	40 (10.4)
Undisclosed *n*	8	–	–
Role, *n*			
Patient	31		–
Parent	–	14	–
Partner	–	3	–
Sibling	–	2	–
Son/daughter	–	2	–
Sibling and daughter	–	1	–
Psychiatrist	–	–	6
Psychologist	–	–	4
Nurse	–	–	10
Ward manager	–	–	6
Support worker	–	–	6
Activity coordinator	–	–	1

### Thematic analysis

The thematic analysis was divided into two domains: exploring participants’ understanding of the ‘carer’ role and how they describe the role they play in hospital settings. Unless otherwise specified, the themes appeared in all three participant types: patients, carers and clinicians. The Appendix contains a summary of the themes and subthemes within these two domains.

### Domain one: who or what is a carer?

It was difficult to find a universally acceptable term for a ‘carer’, as there was diversity in participants’ perceptions of what a ‘carer’ does. To some extent, caring could be seen as an every day part of human relationships. Some carers felt it did not need a label or further recognition, and so they preferred to use everyday terms such as ‘mother’ or ‘father’ instead. However, some carers viewed the caring role as something that is defined by going beyond what a family member would already do. The term ‘carer’ therefore was seen as a title that recognises this. Some clinicians saw it as more of a service term, used between staff only:

‘Like, I would never say, “This is so-and-so's carer” in front of the person.’ (Clinician 32)

‘No, of course not.’ (Clinician 31)

#### Caring in mental health is distinct from physical health

Participants noted how ‘with mental health patients…every bit counts, no matter how small or big’ (Clinician 22) and often, the support was not always clearly defined, but involved ‘being with’ the person. Carers used words like ‘sitting’ ‘minding’ and ‘monitoring’. Through this they said how there was an element of ‘experiencing with’ the person when one is a carer. This was seen as unique to mental health. Participants also described how carers do what staff cannot or do not do. Many participants felt that carers should be rewarded for reducing the pressure on services and for providing the support that nobody else could.

Furthermore, caring in mental health was described as different to physical health in terms of how it ‘crept up’ on people, and one became a carer ‘gradually’, ‘without noticing’. It was a ‘job with no end date’ because the unique nature of the support meant that there was so much uncertainty in the process. It was also felt that carers had little choice in the process. One patient felt that their family being forced to care was akin to a ‘form of slavery’ (Patient 21).

‘Sometimes you could be caring for somebody for such a long time, you don't even know when you started, or when you stopped so to say, “now you're a carer”, it doesn't work like that.’ (Carer 14)

‘Some people don't want to care and they're in a carer role. They're just angry with them, resentful and I've met people who resent being positioned in the family, in the genogram. So location-wise they've got that role and they've got no choice and [Clinician 12: ‘mm’] and others do it because obviously they want to…’ (Clinician 13)

#### Disagreement on when caring begins and ends

Participant groups differed most strikingly in their definitions of when someone can be called a ‘carer’. Clinicians discussed at length the idea that the term ‘carer’ had ‘the idea of some sort of chronicity’ (Clinician 1) and was frequently unnecessary or inappropriate to use it in acute mental health settings.

‘You would use it if you have been yeah, in in mental health services for, you know, quite some time or your relative had an illness that was prolonged for months and months or years then you become a carer but it's not really something in an acute setting.’ (Clinician 5)

Similarly, patients often felt they only had a ‘carer’ when they were unwell, as their fluctuating mental and emotional states meant they experienced fluctuating levels of need. This left carers in the position of always needing to be available but not knowing when their involvement would be welcomed or rejected.

‘Well “carer” is to me like … you're still not well. You're still being cared for. I mean I'm at a stage within my recovery where, I'm not being cared for. I'm caring for myself. So I'm my care – they're just people who are there to help me when I do start falling back and so to me they're not carers, they're support.’ (Patient 24)

‘He [previously] referred to me as a carer, yes, but now where he feels that he's on the road of recovery, he feels that I'm not caring for him as much. But, I am caring for him in what I'm doing for him. I do care for him …I'm having to be the one that has to access the services. I'm the one that has to attend all the meetings [Carer 19: ‘hmm-hmm’] …’ (Carer 18)

Although some family members felt the term ‘carer’ was ‘a bit patronising’ as it implied the patient needed care all the time, many others described the ‘24/7’ nature of caring as all-encompassing and requiring constant monitoring in case the person's symptoms return. One referred to their role as a ‘mind-minder’. Some clinicians also described how they felt carers were the ones who had to ‘deal with it 24/7’ and ‘day in, day out’.

‘We care for them overall…is not just physical or mental. It's everything. [Carer 17: ‘everything’, Carer 18: ‘yeah’] …I think the trouble is that there may be people who think, “It's only when I'm ill in hospital that you have become a carer,” but you are 24/7 worried about it. Worried about when it is going to happen again. [Carer 18: ‘yeah’] …“Has he eaten?” So it's all the time care.’ (Carer 19)

#### Discomfort with dependency

Participants differed in how they conceptualised dependency. Many clinicians and some patients expressed discomfort with the idea of a patient being dependent on a carer.

‘Err it's yeah it kind of implies a bit of feebleness about someone who needs the one cared for long time which isn't necessarily the case and isn't what we're aiming for.’ (Clinician 16)

‘A lot of us have evolved from … I mean, there are probably nurses that do, but most people have moved away from that kind of … You've got a mental health problem, so you are obviously rubbish, you can't do anything, so you must have a carer, kind of thing.’ (Clinician 28)

‘But then I think there's also the other side maybe “carer” like you can't do anything for yourself … you're helpless.’ (Patient 7)

Others viewed dependency differently. They were comfortable with the idea that there might be a temporary period where someone has lost their independence and needs support to look after themselves again. This was often described as a major point of contention with staff.

‘Yeah, a carer is … you know the family, someone you rely on when you can't cook or clean?’ (Patient 27)

‘It's like we have to retrain our loved ones what they used to love to do, what they used to do, we we're teaching them all over again it's like [Carer 7: ‘baby’]. It's like they're babies again [Carer 7: ‘yeah yeah’] but while we're talking to the doctors and people they don't get it [Carer 7: ‘no’] because they just think “he's a big geezer … a big bloke” [Carer 7: ‘mm’].’ (Carer 8)

Although (as above) some clinicians expressed discomfort about disempowering overtones, many carers saw irony in this, as the nature of an involuntary hospital admission itself was viewed as paternalistic by some.

‘They're saying, “They [will] do what they want to do.” Then why do they restrain them? Why do they pin them down? You know?’ (Carer 19)

#### Not all ‘carers’ are caring

A difficult issue specific to mental health was that ‘the one group of people who could be their carers are the problems in some way’ (Clinician 13). This jarred with the idea of calling someone a ‘carer’, as they might be implicated in the person's mental health problems by ‘adding to the stress’ (Clinician 2). Staff did not know always know how to work with this contradiction.

‘… we use it in a quite generic way without really thinking about it but a lot of the time they're not really carers err … a lot of time they don't know enough about the condition to be considered a carer and sometimes they do more harm than good.’ (Clinician 16)

Patients described harm as being misunderstood and being put under pressure to stop ‘playing up’ or ‘attention-seeking’ and that they felt pressure from family members who were pushing for their progress to be ‘two steps further than what it should be’ (Patient 26). They felt strongly that it was their families’ ‘lack of understanding’ that made them feel worse.

‘I don't think they should have any [involvement]. My family they don't understand my illness [Patient 8: ‘mm-hmm’] so I'd rather they don't know anything you know. They don't understand the illness at all.’ (Patient 9)

‘… It's helpful for them to be involved because if they weren't there, we'd have nobody else to help you but at the same time it's hard as well because they don't have the knowledge that they need …’ (Patient 26)

### Domain two: what is a carer's role in hospital?

#### The role of the hospital environment

The hospital environment itself was seen as an important contextual factor that overshadowed all aspects of the patient and carer experience. As described next, participants spoke of the atmosphere and procedures as pertinent factors in determining the role of carers.

##### Frightening atmosphere

All three participant groups’ descriptions of the hospital environment mentioned elements that were unwelcoming and frightening, with one stating it was ‘scary for the relative, just as scary it is for the patients’ (Clinician 17). This was especially the case during the admission process, which was described as ‘a traumatic and chaotic experience for all the involved’ (Clinician 10). During this time, family and friends could be an ‘invaluable’ source of comfort for patients, although this was also a time when both patients and carers were more likely to feel traumatised and in need of information to alleviate concerns. Clinicians described how carers might find it ‘distressing’ to view their relative being restrained or very heavily medicated. For this reason, they sometimes preferred to keep carers away to prevent them from witnessing upsetting scenes. Carers recounted the reassuring impact of ‘very calm staff’ during these times.

‘I was terrified.’ (Patient 25)

‘You're scared of the whole situation, so you don't really talk to anyone, you – you don't wanna talk to staff, ‘cause it's a frightening experience and it takes a while before you can actual feel settled enough to talk to people and…if your carer is someone that's spent a lot of time with you, they know how you are, so you're possibly gonna open up more to them.’ (Patient 24)

‘Especially when it's your first time. I remember my first time [Carer 1: ‘first time’] was a nightmare [Carer 1: ‘nightmare’] [Carer 2: ‘yeah’] yeah nightmare, bad memories.’ (Carer 3)

##### Inflexible systems: wards rounds and the medical model

Moreover, the structure of the hospital system itself was seen as an indirect barrier to the meaningful involvement of carers. Ward rounds were viewed by many participants as the main way families can be involved but also the greatest source of difficulty. Carers described them as inflexible and a source of uncertainty and stress. As most important decisions were made there, carers considered consultant psychiatrists to be the most important people to work with. However, clinicians noted that it is the consultants that often have little time to spend with individual patients and carers.

‘And it can be quite intimidating as well [Carer 11: ‘completely’]. You walk in and everyone's kind of all eyes on me.’ (Carer 3)

‘it's like a panel [Carer 11: ‘yeah’] isn't it?…I still can't get my head round what care co-ordinator, a social worker and somebody else does [Carer 11: ‘mhm’]  ….’ (Carer 14)

‘… other than the psychiatrist, who else needs to be in the room?…I don't actually know why so many people have to be in the room if they don't have actual involvement in the patient's care? ‘Cause otherwise it looks like they're just … it's one of their team's meetings and we're part of the entertainment.’ (Carer 15)

Clinicians in the focus groups were able to critically reflect on the system that they work in, recognising that it was very rigid, with medication frequently at the forefront of discussions. This placed carers’ role on the periphery, as they were often not seen as a core part of the patient's care or the clinical team's routine procedures. Working with carers was seen as a resource-intensive add-on service that was difficult to provide.

‘The service itself doesn't lend itself for you to implement –.’ (Clinician 28)

‘Yes, it's not flexible enough.’ (Clinician 29)

‘ – It's not flexible enough for a carer to access help.’ (Clinician 28)

‘ – Because, we work to a medical model. So the focus is we've got this ward round, we've changed the meds and that's…we'll all sit here, you can walk in, your back is against the wall and you've got 15 min, off you go. If that. Because we've got to talk about meds and everything else…It's chaired by a medic. It's their set time and you suit that.’ (Clinician 26)

‘ – And we call ourselves a client-led service – ’ (Clinician 29)

‘ – Yeah, exactly [laughs].’ (Clinician 28)

##### Patients and clinicians as gatekeepers

Carers were not perceived as having any rights to involvement – the decision was seen as one that either the patient or clinician had to make. Admission was a particularly difficult time as the patient might not have capacity or might feel ‘hostile to family’. Clinicians described how this period was a ‘struggle’ and how difficult it was to ‘balance both needs’ in these situations.

‘It's about them [the patients], exactly.’ (Clinician 32)

‘Yes, so it's their choice and their rights.’ (Clinician 33)

Participants from all three stakeholder groups felt it important to override the patient's wishes in order to act in their ‘best interests’, as carers were seen as a source of contextual information that could facilitate the admission process and as a source of support for patients. Some patients expressed regret that they had excluded carers. However, many still felt strongly that they should always be the ones to decide the level of involvement.

‘That's where the carers come in, doesn't it? Initially the first, second, third day, that's where the carer's voice should be heard more than being pushed aside.’ (Carer 17)

‘I think they – they should be involved even if you're paranoid. If it's in your best interests that these people know then I think they should be…should be informed.’ **(**Patient 30)

‘I want them brought in, then I would bring them in but in ten years I've only brought them in once. I tell them not everything but I tell them … um which stuff's safer.’ (Patient 8)

#### The roles allocated to carers

Within this context, the way carers were described could be broadly allocated to one of three roles: (a) a useful resource, sometimes in need of support themselves; (b) troublemakers, creating a hindrance to everyday clinical procedures or (c) invisibles, having no clear role and not being central to anything. The allocated role largely depended on how clinicians conceptualised the role of carers, as the same carers described being included and valued in some settings and excluded in others.

##### A useful resource, that requires care

Carers could be seen as a useful resource for the healthcare team. One source of their knowledge came from knowing the patient intimately when they were ‘well’ and were able to contrast this with their current mental state.

‘They've gone through the process of you going from being well to getting unwell, so they they're kind of experts around your care and they need to be involved fully with the psychiatrist, the team.’ (Patient 7)

Carers were then able to advocate for the patient during a time that they had difficulty expressing their needs. They were also seen as supporters of symptom monitoring and treatment adherence. Staff described examples of the involvement of carers resulting in a positive impact on patients’ outcomes, and speedier recovery.

Some saw the carer's role as a person who is in need of support themselves. Clinicians described how they saw it as their responsibility to support carers individually, while maintaining the patient's confidentiality. However, often patients’ and clinicians’ descriptions of how to support carers was limited in nature, required consent from the patient, and was largely aimed at supporting the carer to continue caring.

‘I think that calling your family members or a friend or a relative a “carer” is quite good but then there should be a little bit more input in terms of you know supporting them to care for the patient.’ (Patient 20)

‘I think in the case of acute mental health treatment, I think family … need the most reassurance and the most education as well about what's going wrong. They need to have their life – have their mental health right so they can care for someone [else].’ (Patient 1)

##### Troublemakers

Conversely, carers in the groups described how they could just as easily be seen as ‘busybodies’ and ‘troublemakers’ who were ‘overinvolved’ and presented a hindrance to services. Related to this, many carers described the hospital as a place of ‘battle’ that was ‘daunting’ and where they had to ‘fight’ and be ‘pushy’ to be included. This subtheme was particularly pronounced, and discussed at length in the focus groups.

‘Who we care for, our loved ones, they don't realise what we have to go through when confronting professionals. […] you get seen as the trouble maker …So it is a massive battle. Until this day I still get missed off the list for CPA [Care Programme Approach] meetings. Recently, I've just got told the day before, and that was not even from the [team].’ (Carer 18)

‘I used to go each week and ask to go to the ward round and I wasn't allowed to go…'til one day I broke in to one of them.’ (Carer 11)

This notion was supported by patient and clinician examples, who described clinicians intentionally excluding carers with the aim of ‘facilitating procedures’ and protecting patients and themselves from ‘overwhelm’ due to ‘overinvolvement’.

‘In our ward rounds, we don't have family involved at all. We used to but I don't think the consultant liked it. I think it was too much.’ (Clinician 32)

‘…they [carers] intervene too much [Patient 8: ‘mm-hmm’].’ (Patient 9)

‘… there's a cooling off period before the patients settle down. If there's a relationship problem … we will keep them away for a while until such tempers settle down and perhaps they [patients] can explore [if] their presence or involvement will be of any benefit …That is [a] clinical decision, team decision, yes.’ (Clinician 4)

However, some patients viewed the solution to these problems as an increase in involvement and education for carers, so they could more appropriately support their needs. Clinicians and carers also felt that carers would participate with ‘better understanding and less interference’ (Clinician 17) when given information and reassurance.

‘My father was displaced as my nearest relative and I think instead of displacing him they should have given him more education and information and raised awareness about my condition so that maybe he could have come to an understanding make a more informed decision, ‘cause he was saying he doesn't want me to be detained and they said, “okay, we are just gonna displace your nearest relative”, which I found very unhelpful very, very horrible.’ (Patient 7)

‘Yeah, [this time] I was invited to every ward round … everything was explained and that calms your anxiety, if you're given the information.’ (Patient 7)

##### Invisibles

Finally, a less direct type of exclusion was commonly described. Carers often felt ‘invisible’ on the ward and excluded from ward procedures through omission. Unlike the previous theme, not engaging with carers was not necessarily because of intentional exclusion, but because clinicians were unable to see how their role could support carers. This also related to the non-systemic nature of many hospitals, where the main focus of treatment decisions were regarding the patient's symptoms and their medication. Carers were described as having at most a peripheral role in these procedures.

‘I was invisible. You know, I was totally invisible. […] There was never a chance that you could go into there and say, “How was my daughter today?” Because there's no one person to ask. So I'm lost. So I'm just like a visitor, really, when I go to visit. And I was there all the time.’ (Carer 19)

‘When we come to the hospital ward rounds and this and that, they don't really wanna to talk to me sometimes. I'm just sitting there like a dummy [Carer 5: ‘yeah’]. I can't say nothing.’ (Carer 3)

‘You know when you see a mother crying? [Clinician 32: ‘yeah’] It's more emotive than seeing the service user really unwell…’ (Clinician 30)

‘ – I think it's because you know that they're unwell, and there's a reason that they're presenting like that. But then – ’ (Clinician 32)

‘ – And you can do something to help.’ (Clinician 31)

‘Yes. You feel you're actually working to make them feel better. But with the carer, it's like, what can I do to…?’ (Clinician 32)

## Discussion

### Main findings

This was a focus group study exploring patient, carer and clinician views of the role of carers in in-patient mental healthcare. We found some agreement on the types of support carers provide for patients. However, there were differences in opinion between the different stakeholder groups about when someone can be said to be providing care and the point at which someone can ‘justifiably’ be called a carer. Additionally, the hospital setting was seen as both directly and indirectly precluding the involvement of carers. The set-up of the service placed carers in one of three positions: supportive experts that provide collateral information, ‘troublemakers’ who get in the way of ward procedures and, perhaps the most difficult, ‘invisibles’, people who may spend substantial time on the ward but whom staff do not always know how to include in their routine procedures.

There were also multiple differences in opinion regarding the carer role, which might explain why carers often fell into these allocated roles. There was clear disagreement about what constitutes caring and unhelpful behaviour, as was demonstrated in the varying attitudes toward dependency. What some saw as providing essential care, others saw as an impediment to recovery. What emerged was that discomfort with the idea of dependency is not necessarily a universal. Although staff wanted to protect patients from ‘overwhelm’, many patients saw the solution to poor relationships with carers as increased involvement, so they have a better understanding of mental health and can support them more appropriately.

Additionally, although formal definitions of ‘carer’ exist, in reality, there were vast differences in labelling. Family members felt their caring role was all-encompassing in nature, whereas patients and clinicians did not often share this view, and had various personal definitions for when someone can rightly be called a ‘carer’. The disagreement about whether being a ‘carer’ is a constant state, or if it has to be ‘earned’ through caring for someone chronically unwell was linked to mismatched expectations in the clinical setting. It was not always clear if the existence of a ‘carer’ would be acknowledged or accepted, and if this person would be entitled to inclusion, information and support. However, family members themselves reported a lack of choice about their caring role: it felt simultaneously imposed on them and denied from them.

Another major complicating factor were the fluctuations of mental and emotional state in patients in acute treatment. Carers were often left unsure as to how welcome they were as patients changed their minds between wanting to include them and not. This was combined with a range of positive and negative reactions from different staff members towards carers, which compounded the uncertain and stressful nature of the overall experience. Many people were left with the impression that hospital is a ‘frightening’ place or a ‘battleground’.

### Strengths and limitations

The study sample enabled us to explore and compare the views of patients, carers and clinicians from a variety of roles and settings. The diversity in demographics and experiences helped us to identify common experiences across different settings. One potential limitation was the inclusion of people who self-define as a ‘carer’, as they might represent a small proportion of family and friends who are providing support in clinical settings. However, our recruitment strategy included people from a variety of settings beyond carer organisations, such as asking clinicians to share study details with the visitors of people currently in hospital. This ensured that there was diversity among participants in terms of their understanding of the ‘carer’ role.

Overall, while focus groups are a good method for generating ideas, they are not ideal for the in-depth exploration of topics. This study may be viewed as a starting point into more in-depth qualitative enquiry into this area, particularly as this field has a lack of patient perspectives. Finally, this study mainly focused on in-patient treatment, and there might be other complicating factors that have an impact on peoples’ experiences in other settings. Further discussion of strengths and limitations may be found in our linked article.^8^

### Interpretation and comparison with literature

Previous literature highlights the difficulties experienced by families in the clinical setting. Jankovic and colleagues mapped out carers’ experiences and found that difficulties begin to arise long before reaching the hospital admission stage.^7^ This might explain some of the discrepancies in participant views on when someone can be called a ‘carer’. For carers, the less visible process of monitoring to prevent relapse might be experienced as a constant state, not just confined to when the person is unwell. Furthermore, the process leading to admission is often described as traumatic for the family members themselves, resulting in them needing higher levels of information and reassurance, but being unsure if they will receive this, or face exclusion or invisibility.

Studies of carer perspectives describe how they feel that confidentiality is used by clinicians as a reason to exclude them in in-patient settings.^11^ Wilkinson & McAndrew describe families’ feelings of powerlessness that can arise from being excluded and feeling invisible in in-patient settings.^12^ By including clinicians and patients themselves in these focus groups, we demonstrate some of the reasons why carers might be excluded beyond the desire to protect patient confidentiality. In some cases, there appears to be a fundamental clash of values in terms of what is best for the patient. The patient voice itself, however, is not always included in these decisions, as illustrated by examples of family members being excluded to protect patients from overwhelm or because the carer disagrees with the treatment plan.

Each type of exclusion requires a different approach to address it. Intentional exclusion might be avoided through increased communication at the outset. This might include efforts to find common goals and values during the treatment process, or by addressing families’ underlying needs for acknowledgement and reassurance. However, unintentionally leaving families out due to not considering them as central to ward procedures might be a more difficult, systemic issue to address.

The hospital setting being a barrier in itself is usually discussed in terms of the individualistic, non-systemic nature of the setting.^13,14^ Our study specifies some of the most difficult aspects, and why it may be such a challenge to overcome them. The centrality and time-limited nature of ward rounds, for example, emerged as a frustration for all stakeholders. Our linked studies specify some of the practicalities of what could be done to overcome some of these organisational barriers.^6,8^ Frameworks such as the Triangle of Care^15^ or intervention models such as SYMPA (systems therapy in acute psychiatry)^16^ or Family Intervention^17^ can also provide some structure and guidance to this process.

Finally, Landeweer and colleagues highlight differences in what patients, carers and clinicians view to be barriers to family involvement. They suggest these discrepancies are the result of differences in their underlying beliefs and values.^18^ This study illustrates that indeed, even the definition of ‘carer’ is not necessarily agreed among all stakeholders. The reasons for this might be because of different motivations and belief systems about what mental health is and what treatment should entail.^4^ While all parties might view the patient's independence as the ultimate aim, the route to this and the speed at which it happens might not be universally agreed, resulting in conflict in the acute setting.

### Implications for practice

The role of families in hospital settings is not universally agreed. They can present a variety of needs ranging from basic information, emotional support or collaboration to support the patient's treatment. This can pose difficulties for clinicians, who describe the conflict of having to attend to the individual patient while trying to best manage the needs of carers. This is dealt with in different ways, as some choose to work more closely with families and others exclude them from ward procedures. Families therefore face strong uncertainty in the in-patient process, not knowing if they will be welcomed, supported, ignored or excluded.

A further complicating factor emerged that not all clinicians viewed family members as carers and did not see it as their role to include them in clinical procedures. This may be related to what the fundamental purpose of a hospital is perceived to be. If it is solely to attend to the presenting symptoms of a patient, the presence of additional family members will indeed be seen as a hindrance to ward procedures. If a broader, systemic view is taken, those same family members might be conceptualised as major members of the patient's social network, whose presence can be beneficial, whether they are ‘carers’ or not. In reality, many clinicians placed themselves somewhere between both of these views, depending on the ‘ideal’ and ‘realistic’ service they could provide on a given day. However, this inconsistent approach might compound the high level of uncertainty already present in this setting. Structured procedures to routinely identify and support carers might alleviate some of the difficulties described above.

Additionally, as acknowledged by all three participant groups, patient choice is important, but it does not preclude meaningful interactions with carers. Although it must be acknowledged that many ward procedures and confidentiality rules are not set-up to favour carer involvement, positive examples of other ways of engaging carers demonstrated that it is still possible to improve upon patient, carer and clinician experiences in the in-patient setting.

Overall, this study highlights the importance of clarity when considering the ‘carer’ role, as misunderstandings can have the potential to have a negative impact on patient, family and staff experiences. As demonstrated by participant examples, excluding carers might appear to help clinical procedures in the short term, but could create more divisions between patients, carers and clinicians in the long term. Establishing wishes and expectations at the beginning of admission might be one way of opening up the potential for communication and reducing the likelihood that a patient or carer feels they have not been listened to. Finally, giving clinicians the space to have open conversations and critically reflect on core fundamentals such as the role of carers in their work might help them to problem-solve, and decide how to adapt their approach to carer involvement within their own local context.

In conclusion, there is no single agreed definition of ‘carer’. This conflict in how a carer is viewed has the potential to have a major impact on their experience in in-patient settings. The implementation of carer involvement initiatives should incorporate addressing this fundamental aspect. Overall, it could be concluded that there needs to be clearer agreement about the role of carers in hospital settings, as they fluctuate between being perceived of as important resources, passive visitors or adversaries.

## Data Availability

All authors had access to the study data for the duration of data collection and analysis. A.D. and S.P. have ongoing access to the transcripts.

## Appendix

### Domains, themes and subthemes

**Table:**

Domain	Theme	Subtheme
Who or what is a carer?	Caring in mental health is distinct from physical health	
	Disagreement on when caring begins and ends	
	Discomfort with dependency	
	Not all ‘carers’ are caring	
What is a carer's role in hospital?	The role of the hospital environment	Frightening atmosphere
		Inflexible systems: wards rounds and the medical model
		Patients and clinicians as gatekeepers
	The roles allocated to carers	A useful resource, that requires care
		Troublemakers
		Invisibles

## References

[1] Pitschel-Walz G, Leucht S, Bäuml J, Kissling W, Engel RR. The effect of family interventions on relapse and rehospitalization in schizophrenia - a meta-analysis. Schizophr Bull 2001; 27: 73–92. 11215551 10.1093/oxfordjournals.schbul.a006861

[2] National Collaborating Centre for Mental Health (UK). Psychosis and Schizophrenia in Adults: Treatment and Management. NICE, 2014.

[3] Department of Health and Social Care. Recognised, Valued and Supported: Next Steps for the Carers Strategy. HMSO, 2010 (https://www.gov.uk/government/publications/recognised-valued-and-supported-next-steps-for-the-carers-strategy).

[4] Dirik A, Sandhu S, Giacco D, Barrett K, Bennison G, Collinson S, Why involve families in acute mental healthcare? A collaborative conceptual review. BMJ Open 2017; 7: e017680. 10.1136/bmjopen-2017-017680 PMC5623469 28963308

[5] Ince P, Haddock G, Tai S. A systematic review of the implementation of recommended psychological interventions for schizophrenia: rates, barriers, and improvement strategies. Psychol Psychother 2016; 89: 324–50. 26537838 10.1111/papt.12084

[6] Eassom E, Giacco D, Dirik A, Priebe S. Implementing family involvement in the treatment of patients with psychosis: a systematic review of facilitating and hindering factors. BMJ Open 2014; 4: e006108–8. 10.1136/bmjopen-2014-006108 PMC4187461 25280809

[7] Jankovic J, Yeeles K, Katsakou C, Amos T, Morriss R, Rose D, Family caregivers’ experiences of involuntary psychiatric hospital admissions of their relatives – a qualitative study. Harrison BJ, editor. PLoS One 2011; 6: e25425–7. 22022393 10.1371/journal.pone.0025425 PMC3192057

[8] Giacco D, Dirik A, Kaselionyte J, Priebe S. How to make carer involvement in mental health inpatient units happen: a focus group study with patients, carers and clinicians. BMC Psychiatry 2017; 17: 1–13. 28320376 10.1186/s12888-017-1259-5 PMC5359804

[9] Ritchie J, Lewis J, Nicholls CM, Ormston R. Qualitative Research Practice. Sage, 2014.

[10] Braun V, Clarke V. Using thematic analysis in psychology. Qual Res Psychol 2006; 3: 77–101.

[11] Gray B, Robinson C, Seddon D, Roberts A. “Confidentiality smokescreens” and carers for people with mental health problems: the perspectives of professionals. Health Soc Care Community 2008; 16: 378–87. 18194286 10.1111/j.1365-2524.2007.00748.x

[12] Wilkinson C, McAndrew S. “I‘m not an outsider, I'm his mother!” A phenomenological enquiry into carer experiences of exclusion from acute psychiatric settings. Int J Ment Health Nurs 2008; 17: 392–401. 19128286 10.1111/j.1447-0349.2008.00574.x

[13] Stanbridge R, Burbach F. Establishing family inclusive acute inpatient mental health services: a staff training programme in Somerset. J Fam Ther 2009; 31: 233–49.

[14] Fadden G, Birchwood M, Lefley H, Johnson DL. British models for expanding family psychoeducation in routine practice In Family Interventions in Mental Illness: International Perspectives (eds HP Lefley, DL Johnson): 25–42. Praeger, 2002.

[15] Worthington A, Rooney P. The Triangle of Care; Carers Included: A Guide to Best Practice in Acute Mental Health Care. Carer's Trust, 2010 (https://carers.org/downloads/resources-pdfs/triangle-of-care-england/the-triangle-of-care-carers-included-second-edition.pdf).

[16] Schweitzer J, Ginap C, Twardowski Von J. Training psychiatric teams to do family systems acute psychiatry. J Fam Ther 2007; 29: 3–20.

[17] Kuipers L, Leff J, Lam D. Family Work for Schizophrenia. RCPsych Publications, 2002.

[18] Landeweer E, Molewijk B, Hem MH, Pedersen R. Worlds apart? A scoping review addressing different stakeholder perspectives on barriers to family involvement in the care for persons with severe mental illness. BMC Health Serv Res 2017; 17: 1–10. 28506296 10.1186/s12913-017-2213-4 PMC5433083

